# Quantifying international human mobility patterns using Facebook Network data

**DOI:** 10.1371/journal.pone.0224134

**Published:** 2019-10-24

**Authors:** Spyridon Spyratos, Michele Vespe, Fabrizio Natale, Ingmar Weber, Emilio Zagheni, Marzia Rango

**Affiliations:** 1 Knowledge Centre on Migration and Demography, Joint Research Centre, European Commission, Ispra, Italy; 2 Qatar Computing Research Institute at Hamad Bin Khalifa University, Doha, Qatar; 3 Max Planck Institute for Demographic Research, Rostock, Germany; 4 Global Migration Data Analysis Centre, International Organization for Migration, Berlin, Germany; Tel Aviv University, ISRAEL

## Abstract

Quantifying global international mobility patterns can improve migration governance. Despite decades of calls by the international community to improve international migration statistics, the availability of timely and disaggregated data about long-term and short-term migration at the global level is still very limited. In this study, we investigate the feasibility of using non-traditional data sources to fill existing gaps in migration statistics. To this end, we use anonymised and publicly available data provided by Facebook’s advertising platform. Facebook’s advertising platform classifies its users as “lived in country X” if they previously lived in country X, and now live in a different country. Drawing on statistics about Facebook Network users (Facebook, Instagram, Messenger, and the Audience Network) who have lived abroad and applying a sample bias correction method, we estimate the number of Facebook Network (FN) “migrants” in 119 countries of residence and in two time periods by age, gender, and country of previous residence. The correction method estimates the probability of a person being a FN user based on age, sex, and country of current and previous residence. We further estimate the correlation between FN-derived migration estimates and reference official migration statistics. By comparing FN-derived migration estimates in two different time periods, January-February and August-September 2018, we successfully capture the increase in Venezuelan migrants in Colombia and Spain in 2018. FN-derived migration estimates cannot replace official migration statistics, as they are not representative, and the exact methods the FN uses for classifying its users are not known, and might change over time. However, after carefully assessing the validity of the FN-derived estimates by comparing them with data from reliable sources, we conclude that these estimates can be used for trend analysis and early-warning purposes.

## Introduction

In this article, we investigate the use of data from non-traditional data sources for estimating international human mobility patterns. Non-traditional data sources provide data that are generated actively or passively by internet users or devices. In this study, we take advantage of anonymised and publicly available data provided by Facebook’s advertising platform. This platform enables advertisers to run advertisements targeted at users of Facebook’s family of applications and services, which include Facebook [1], Instagram [2], Messenger [3], and the Audience Network [4]; and which we hereafter refer to as the “Facebook Network” (FN). These apps are widely used in many parts of the world, and, as of November 2018, collectively had 2.1 billion monthly active users. Social media accounts are typically linked to various types of information, including users’ present and past geographical locations and demographic characteristics, such as age and gender. In particular, we take advantage of the profiling of FN users by their present and past place of residence to explore the characteristics of migrants. Measuring international mobility at a global scale is essential for enhancing our understanding of migration-related phenomena. This knowledge can, in turn, improve migration governance by supporting evidence-based policy-making and informing the public debate on migration. The need to improve migration data is mentioned in the first objective of the Global Compact for Safe, Orderly and Regular Migration, adopted by the UN Member States in December 2018: “Collect and utilize accurate and disaggregated data as a basis for evidence-based policies”.

The main source of international statistics on migration is the United Nations Department of Economic and Social Affairs, which provides global estimates of international migrant stocks disaggregated by origin and sex, with the most recent estimates referring to the year 2017 [5]. The OECD provides information on international migrant stocks and flows, student mobility, and aspects of migrant integration in the OECD member states. The statistics provided by international bodies are based on national statistics that mainly come from population censuses, but also from household surveys and administrative data sources. Although efforts have been undertaken in recent years to improve international statistics on migration, several significant challenges remain. The first challenge is related to the coverage of data. While statistics on migratory flows are regularly collected for OECD and EU countries of destination, there is a lack of even basic information about the number of people emigrating and immigrating on an annual basis for a large share of the world’s developing countries. The second issue is related to a lack of detail about the characteristics of the migrant population. When considering the impacts of migration on the destination and the origin countries, the integration needs of the migrants, and the policy implications of migration, having an awareness of the very large differences between different forms of migration is essential. Recognising these distinctions requires a deeper understanding not only of the basic demographic characteristics of migrants, such as age and gender; but of migrants’ educational levels, socio-economic status, reasons for migration, and legal status in the destination country. The third issue is linked to the timeliness of data. Especially since the 2015 migration crisis in Europe, policy-makers have been expressing an urgent need for data that will allow them to anticipate emerging trends and establish early warning systems for migration. However, because of the complexity of the processes and the resources required for the collection, quality control, and dissemination of such data from the national to the international level, frequent updates of these statistics have not been possible.

In recent years, several researchers have explored the option of using non-traditional data sources to study international human mobility patterns and migration. Zagheni and Weber [6] proposed a general framework for extracting demographic information from non-representative internet data. Relevant work has been carried out by Zagheni, Weber, and Gummadi [7], and Spyratos et al. [8], who proposed two methodologies for estimating the stock of Facebook/FN “migrants/expats” in the US and the EU, respectively. Recently, Zagheni et al. [9] developed a Bayesian hierarchical model that uses both Facebook and official statistical sources for generating up-to-date estimates of migrant stocks. Other non-traditional data sources that have been used for quantifying international human mobility include Twitter [10–13], Yahoo [14,15], Google [16], LinkedIn [17,18], Flickr [19], and Call Detail Records (CDR) from mobile communication operators [20]. A report prepared for the European Commission suggested that it may be feasible to use non-traditional data to estimate migration flow trends in a timely manner [21]. Finally, the potential use of non-traditional data sources for the analysis of migration-related issues was explored in a dedicated workshop organised by the European Commission Knowledge Centre on Migration and Demography (KCMD) and IOM’s Global Migration Data Analysis Centre (GMDAC) [22].

The non-traditional data sources used to complement official statistics on migration have advantages and limitations. Among the advantages of these sources are their richness of detail, their high degree of spatial or temporal resolution, and their coverage of hard-to-reach segments of the population and areas of the world. Another important advantage these sources offer is that they are able to capture forms of migration and international mobility that are usually not represented in official statistics on migration, such as temporary migration and circular migration [20]. A main disadvantage of these sources is that they are not primarily designed to monitor migration. Even if their sample sizes cover nearly the entire population, these sources cannot be considered statistically based and fully representative. Moreover, because the definitions and the data collection and processing methods used in these sources are not standardised and are often not precisely stated, adapting them for the study of migration is not easy. Finally, another non-trivial limitation of using these sources lies in the availability of the data for research purposes, which is constrained by the fact that these data are owned by private companies.

While the FN data explored in this paper are from a non-traditional data source, they cannot be considered big data. Instead of analysing directly raw and individual records of personal information, we rely on pre-aggregated figures that describe the population based on several possible profiling attributes. These data are more similar to classical population statistics, as Facebook’s advertising platform has taken on the task of providing aggregates from the wealth of individual and personal information stored in their databases. A considerable advantage of using aggragated data is that it allows us to bypass all the technical and legal challenges that arise when dealing with large volumes of personal data. However, the questions of how these aggregate figures are being produced, and how representative they are with respect to the population of migrants, remain open. This is because there is a lack of information about the proprietary algorithms that social media companies use to process personal data and classify users based on their behaviour. Data from social media do not represent all societal groups equally. People with different age, sex, income, and racial characteristics use social media applications to different degrees [23]. In addition, the penetration rate of social media within a specific demographic group might change from one year to another, and from one country to another. The reliability of self-reported information on social media is also questionable. Facebook has estimated that in the fourth quarter of 2018, fake accounts and duplicate accounts made up about 5% and 11% of its worldwide monthly active users, respectively [24]. The use of data from social media raises various privacy and ethical concerns. For instance, openly available location data from social media, even in aggregated form, may be used to track the movements of vulnerable groups such as displaced persons not only by humanitarian organisations, but by criminal organisations. Furthermore, these data may be used to monitor the behaviours of users in ways that could encroach on their civil liberties. The analysis of social media data also poses serious data protection issues, since users often have limited awareness of what data social media companies collect, process, and share with third parties [25]. According to a survey of Facebook users conducted by Hiltin and Rainie [26], 58% of respondents who were informed that Facebook’s advertising platform has been categorising them according to their interests were not very or not at all comfortable with Facebook compiling this information.

This paper benchmarks the methodology of Spyratos et al. [8] against 55 countries of current residence, and demonstrates the potential for using FN statistics for estimating temporal changes in the stocks of FN users who have lived abroad. The methodology we use does not aim to provide FN estimates of people who have lived abroad as an alternative to official migration statistics. As we explain in more detail in the next section, the definition of users who have “lived abroad” employed by Facebook’s advertising platform is not fully known, and is not equivalent to the definition of an international migrant used in official statistics. We correct for the potential bias in the FN user population by creating separate estimates of migration that can complement official statistics on migration by providing additional input on the more general phenomenon of international human mobility.

This article is organised as follows. The data section presents and describes the FN user statistics and the official migration statistics we draw upon in this study. The methodology section describes the data preparation process and the methodology used to estimate the number of FN “migrants” in the real population based on the number of FN users who are classified as having lived abroad, by age and gender. The results section presents a comparison between the official migration statistics and estimates of FN “migrants”, as well as a comparison between FN “migrant” estimates collected in two times periods, January-February 2018 and August-September 2018. A discussion of the results is provided and the conclusions are outlined in the final section.

## Data

### Facebook Network user statistics

In this study, we chose to use the Facebook Network as the non-traditional source of data for estimating “migrants”, because it is, to the best of our knowledge, the only widely used social network that classifies its users based on their previous residence, and provides these estimates on a publicly accessible advertisement platform. Through its advertising platform [27], Facebook allows customers to design targeted advertisements and publish them on the Facebook family of apps and services, which we refer to in this study as the FN. The FN includes Facebook, Instagram, Messenger, and the Audience Network. Advertisers can select the characteristics of the FN users they want to reach with their campaign. After a customer has selected the characteristics of the target audience, the advertising platform provides information on the number of its daily active users (DAU) and monthly active users (MAU) who match these criteria as an indication of the potential audience that can be targeted with the advertisement campaign. Among the characteristics that can be used as search criteria are variables that refer to the users’ place of residence, age, gender, interests, consumer behaviour, and–particularly relevant for our purposes–previous country of residence. For example, a prospective advertiser can get an estimate of the number of FN users who are currently living in France, are male, are between the ages of 15 and 20, and who were previously living in Brazil. These DAU and MAU estimates are not designed to match census population data, and are affected by how many accounts each person has, how many visitors there are in a particular geographic location at a given time, and user-reported information.

Facebook’s advertising platform classifies some of its users as “lived in country X”; or, until October 2018, as “expats of country X”. This classification is provided for the 89 countries of previous residence listed in S1 Table. In Facebook’s advertising platform documentation, users who have “lived in country X” are defined as “people who used to live in country X and now live abroad”. Due to the lack of detailed documentation, it is not clear what specific criteria Facebook’s advertising platform is using to establish the previous country of residence of each FN user. Despite this lack of documentation, we can infer based on the literature produced by researchers who worked at Facebook [28] that two factors play a key role in the identification of expats at Facebook. The first factor is the self-reported “current city” and “hometown” in the list of “places you have lived” that people fill in for their Facebook profile. The second factor is the network structure of friendships (e.g., having at least two Facebook friends in the home country and two Facebook friends in the destination country). Herdagdelen et al. [28], while working at Facebook, generated estimates of migrants based on the set of variables we just described. In their publication, they provided a ranking of the top 11 immigrant communities in the US on Facebook according to their country of origin (Mexico, India, Philippines, Puerto Rico, El Salvador, Dominican Republic, Guatemala, Canada, Honduras, Cuba, and Colombia). This ranking is almost exactly identical to the one provided by Facebook’s advertising platform, as reported in [7]. Based on a non-representative survey with a small sample size, Spyratos et al. [8] concluded that Facebook infers users’ places of current and previous residence from geo-referenced information, as well as from self-reported information. It appears that the FN uses geo-referenced information apart from the user’s self-declared “hometown”, given that 13 participants in the Spyratos et al. [8] survey were classified correctly as having lived in their home country, even if they did not provide information on their hometown. Moreover, another three participants in the survey were classified based on their country of previous residence, and not on the country of the hometown they listed in their profile. Yet another participant was classified as having lived in both the country of his/her stated hometown and his/her country of previous residence. Although Facebook might identify “migrants” in many different ways, including using very complex algorithms that are evolving over time, we are confident that “hometown”, “current city”, and “other places lived”, as well the network structure of Facebook friendships, are among the key components of the FN’s identification process.

In this paper, we use the term “FN migrants” to refer to FN users who have lived in a country other than their current country of residence. Facebook’s definition of users who “lived in country X” is quite broad, and is based on a rather loose concept of migration. Thus, it might capture a wide range of international mobility forms–i.e., temporary, permanent, or circular migration–with no reference to a user’s citizenship, country of birth, or legal status. The definitions used in international statistics for reporting data on international migrants [29] are generally based on three separate criteria: whether a person was born in a country other than the country where s/he is currently living (country of birth), whether a person has a nationality (citizenship) other than that of the country where s/he is currently living, and whether a person is residing in a country other than the country where s/he was previously living (previous residence). While the definition Facebook uses is not compatible with any of the definitions of an “international migrant” used by the United Nations, it is most similar to the official definition of previous residence. However, since we lack access to international migrant stock data by previous residence, the approach we rely on in this study (which we describe in the next section) is to compare the FN-derived estimates with official statistics describing the country of birth. It should also be noted that while official statistics are normally disaggregated by sex based on individuals’ biological characteristics, the FN data are disaggregated by gender based on how users identify themselves.

The datasets that we collected from Facebook’s Marketing Application Programming Interface (API) cover two different time periods, and both the total population in each country and the FN migrants. The dataset *FN_mig_raw*^*i*^_*a*,*g*,*o*,*d*,*p*_, in Eq 1, is the number of the FN’s MAUs whose current country of residence is *d*, in age group *a*, of gender *g*, and in time period *p;* and who are classified by Facebook’s advertising platform as having lived in country *o*. To slightly expand the coverage of the population, we chose to use statistics about all of the FN users, and not to restrict the data collection to users of one or more FN applications like Facebook or Instagram. The dataset *FN_pop_raw*_*a*,*g*,*c*,*p*_ in Eq 2 indicates the total number of the FN’s MAUs, including both migrants and non-migrants, whose current country of residence is *c*, who are in age group *a*, who are of gender *g*, in time period *p*. Due to the API rate limits of approximately one API call every 10 seconds, the first data collection period, *p = 1*, for both the *FN_mig_raw*^*i*^_*a*,*g*,*o*,*d*,*p*_ and *FN_pop_raw*_*a*,*g*,*c*,*p*_ datasets lasted from 30/1/2018 to 26/2/2018. On 26 February, the minimum MAU response of Facebook’s marketing API to queries was increased from 20 to 1000 users. This change in the minimum MAU response is intended to protect the privacy of the users, and it means that as long as the total number of users in a selected group is lower than 1000, Facebook advertising platform will only report that there are fewer than 1000 users with the selected characteristics. The second data collection period, *p = 2*, lasted from 13/08/2018 to 19/9/2018. In this study, we used numbers of MAU instead of numbers of DAU, since the DAU count is affected by fluctuations in the FN’s weekly usage patterns over such long data collection periods.

$$FN\_mig\_raw^{i}{}_{a,g,o,d,p}$$ (1)

$$FN\_pop\_raw_{a,g,c,p}$$ (2)

*where a = age in five-year age groups* ϵ *[15–19, 20–24, 25–29, 30–34, 35–39, 40–44, 45–49, 50–54, 55–59, 60–64]*

*g = gender* ϵ *[Male*, *Female]*

o = country of previous residence; see S1 Table (89 counties).

d = country of current residence; see S2 Table (120 countries)

*p = data collection period 1*: *(30/1–26/2/2018)*, *2*:*(*13/08-19/9/2018)

*c = country* ϵ *o* ∪ *d*

**Fig 1:**
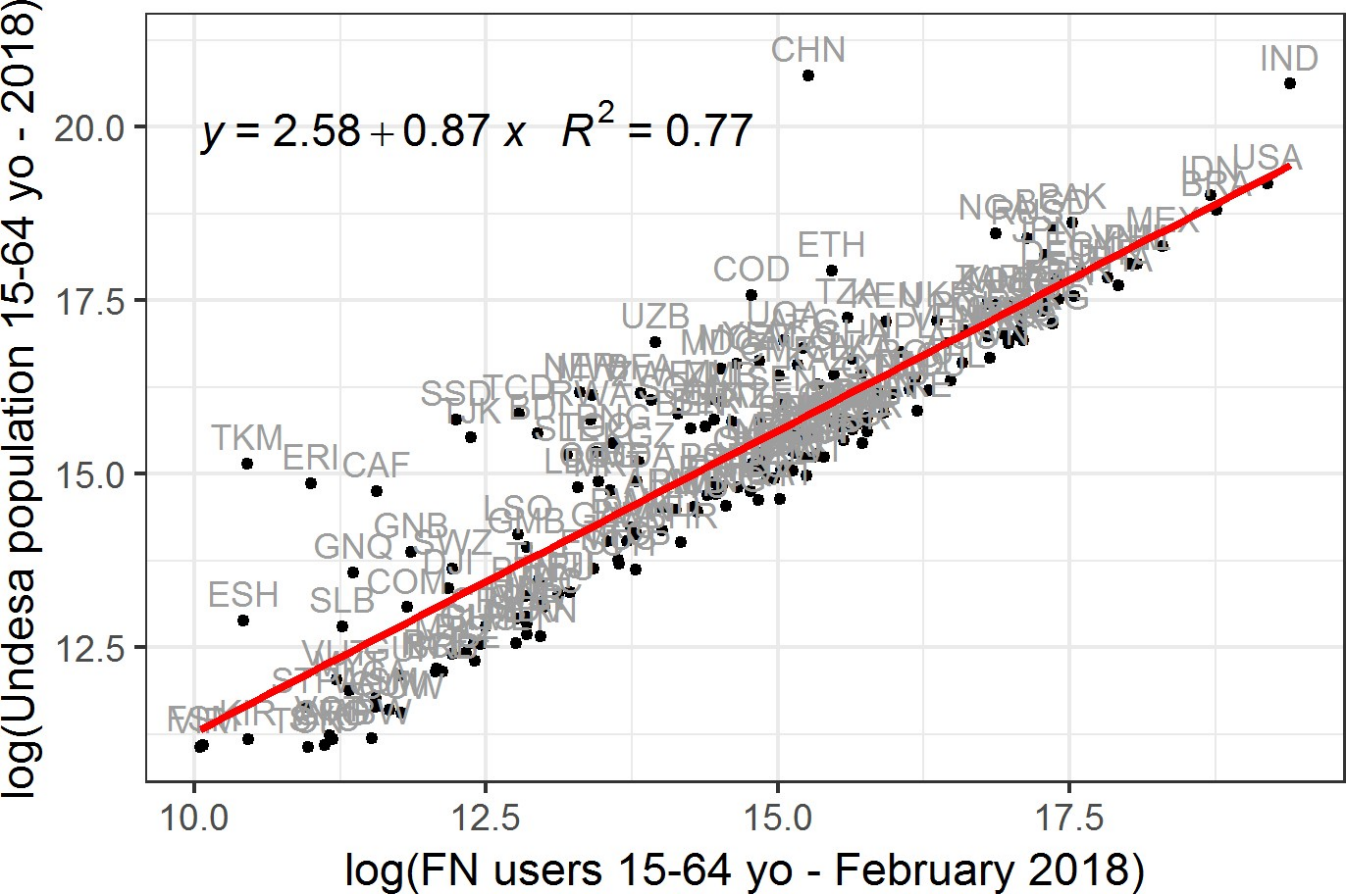
Log-log linear regression between the 2018 population aged 15–64, as estimated by UNDESA; and the number of FN users aged 15–64 for each country in February 2018.

The first step in our analysis was to evaluate the level of coverage of the total population of FN users in each country with respect to the population reported in official statistics, independent of their migratory status. As Fig 1 shows, the level of FN usage varied considerably across countries. In addition, even within the same country, FN usage varied across time. For instance, the number of FN users aged 15 to 64 in Italy was 32,440,000 in February 2018, but was 31,970,000 in October 2018. The FN penetration rate in any given country is mainly affected by three types of factors. First, the rate is influenced by technological, economic, or political constraints, such as limited or costly internet access, or restrictions in access to the FN in countries like China. Second, the rate is affected by socio-cultural factors. For example, in countries with conservative gender norms, women may be less likely to use social media than men [30]. Third, the rate is influenced by the popularity of alternative platforms. For example, the most popular social networking site in Russia is not Facebook, but VKontakte.

**Fig 2:**
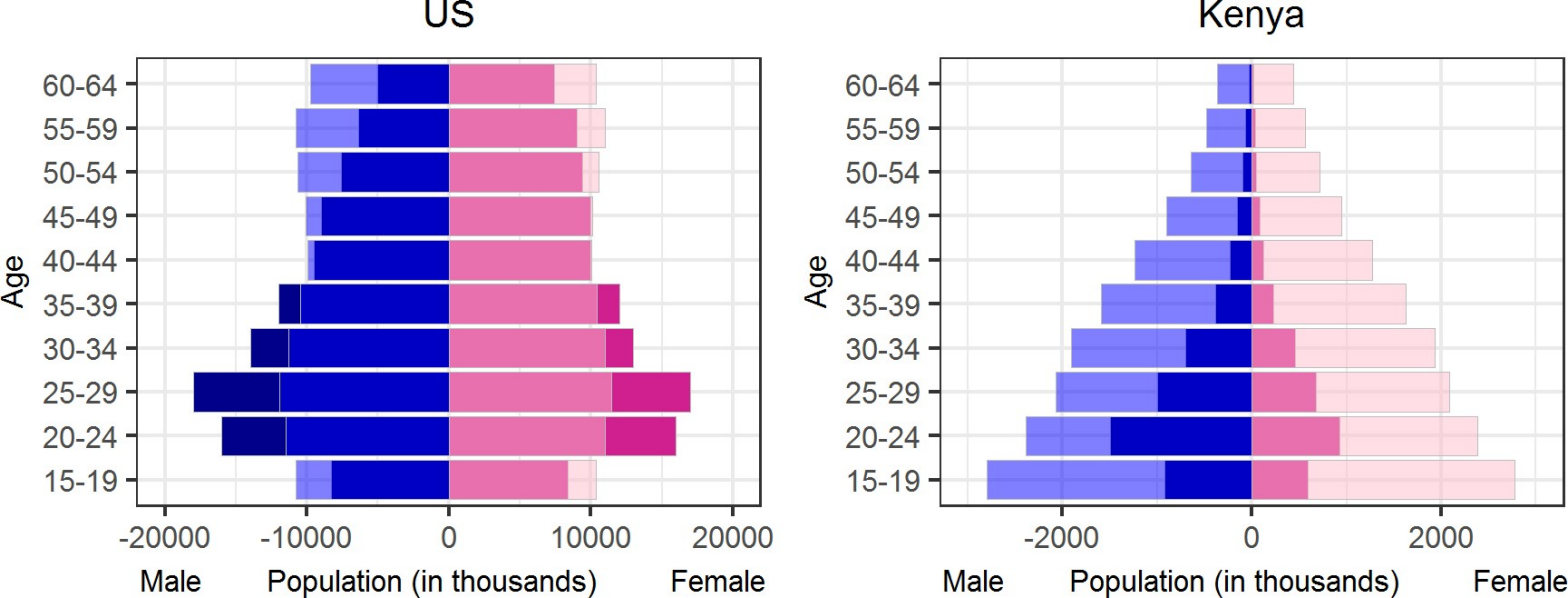
UNDESA population and FN users in the US and Kenya by age and sex/gender. Light shading = UNDESA population, mid shading = UNDESA & raw FN users, and dark shading = raw FN users.

As Fig 2 indicates, the probability of having a FN account also varies based on gender and age. For example, in Kenya, males are more likely to be FN users than females, while in the United States of America (US) females and males are just as likely to be FN users. Moreover, FN usage is extremely low among people aged 35 or older in Kenya, but this is not the case in the US. The number of FN users in a country who belong to a given demographic group can be higher than the actual number of residents in the same group based on the UNDESA population statistics. This over-representation may have occurred because FN users have multiple unlinked FN accounts.

### Official statistics

Besides evaluating the level of coverage of the entire population by country, age, and gender, we relied on official statistics about international migrant stocks. These statistics can be used to determine the degree to which a “FN migrant” user assimilated to the FN usage patterns of the country of current residence, and for benchmarking the results of our estimates of “FN migrants”. Global migrant stock statistics are available based on country of birth or country of citizenship, but not by country of previous residence. In this study, we compared the FN-derived estimates with migration statistics that mainly cover the foreign-born population, rather than foreign citizens. We assume that Facebook’s advertising platform’s classification of users as having “lived in country X” mostly refers to users who are living in a country other than their country of birth, rather than to users who are living in a country other than the country of their citizenship. This assumption is made because Facebook’s advertising platform mainly relies on the country of a user’s hometown to classify a user as having lived in that country [8], and this is likely to be the user’s country of birth. In this study, we mainly draw on three sources of migration statistics, which are described in detail below.

The American Community Survey (ACS) is an annual survey conducted by the US Census Bureau [31]. The ACS collects information about the demographic characteristics of individuals and households residing in the US. Of interest to this study is the ACS information related to the population by age, sex, and country of birth, presented in Eq 3. The ACS migration statistics used in this study are based on a sample interviewed between 1 January and 31 December 2017.

Eurostat [32] provides migration statistics for 18 EU and the four European Free Trade Association (EFTA) countries, disaggregated by age, sex, and country of birth. The Eurostat dataset is described in Eq 4, and is named “Population on 1 January 2018 by age group, sex and country of birth”.

Due to the absence of updated migration statistics disaggregated by age, sex, and origin at the global level, we combined the recent UNDESA [5] migrant stock estimates, which do not provide information about the age structure of the migrant population, with migration statistics provided by the OECD [33]. The UNDESA [5] dataset, entitled *International migrant stock by destination and origin*, provides estimates of international migrant stocks at mid-year (1 July 2017), disaggregated by sex, country of origin, and destination. The UNDESA estimates are mainly based on data from national population censuses, but are also based on population registers and nationally representative surveys for some countries.

The OECD provides two migration datasets disaggregated by age, sex, country of origin and destination for the reference years 2010/11, which we merged. The first is the *Database on Immigrants in OECD Countries* (DIOC), and the second is the *Database on Immigrants in OECD and non-OECD Countries* (DIOC-Extended).

To obtain recent estimates of migrants aged 15–64 in countries not included in the Eurostat or the ACS statistics, we assumed that the ratio of male or female migrants aged 15–64 to migrants of all ages remained the same from 2010/11 to 2017. As described in Eq 5, we estimated the *un*_*a = 15–64*,*s*,*o*,*d*_ by multiplying the percentage of migrants of sex *s*, origin *o*, in destination *d* who belong to the age group 15–64 compared to all ages 0+, based on OECD statistics, by the number of migrants of the same sex s, origin *o* in destination *d*, as estimated by UNDESA for 2017. The combination of the OECD and UNDESA datasets resulted in a new dataset *un*_*a = 15–64*,*s*,*o*,*d*_, which includes migrant stock statistics for 34 countries.

$$acs_{a}{}_{,s,o,d}$$ (3)

$$eur_{a}{}_{,s,o,d}$$ (4)

$$un_{a=15-64,s,o,d}=UNDESA\_mig_{a=0+,s,o,d\;*}\left(OECD\_mig_{a=a=15-64,s,o,d/}OECD\_mig_{a=0+,s,o,d}\right)$$ (5)

*where a = age*

*s = sex [Male*, *Female]*

o = countries of birth

d = country of residence

The reference migration statistics used in this study, as described above, refer to a total of 55 countries of destination (S2 Table). For the EU countries for which Eurostat migration statistics were not available, such as Germany and France, we used UNDESA/OECD statistics. Both the Eurostat and the ACS data refer to the foreign-born population. The UNDESA/OECD estimates equally refer to the foreign-born population in all the 34 countries of destination except two, Botswana and Japan, where the migrant stock statistics identify foreign citizens.

## Methodology

### Preparation of the Facebook Network data

In the FN data preparation phase, we proceed as follows: first, we cleaned the FN data; second, we estimated the lower and upper bounds of Facebook’s API rounded responses; and, third, we estimated the FN penetration rates. All of the data from Facebook’s Marketing Application Programming Interface were provided to us in a fully anonymised format. To the best of our knowledge, this research complies with the Facebook terms of service.

First, during the data cleaning phase, we excluded FN migrant user statistics from four countries out of the 89 countries of previous residence listed in S1 Table, and from one country of out of 120 countries of current residence listed in S2 Table. FN users who are classified as having lived in Greece (formerly “Expats–Greece”) were excluded from our analysis because the FN highly underestimates their number, which is probably due to an error of the Facebook classification algorithm. As of August 2018, there were only 9600 FN users worldwide who were classified as having lived in Greece, whereas according to UNDESA [5] estimates, there were 993,000 individuals who were born in Greece and were living outside of the country in 2017. China was excluded from our analysis as both a country of current residence and a country of previous residence because access to the FN is blocked in China. We also excluded FN users who had lived in Cuba and Monaco because the data required for the estimation of the FN penetration rates in these countries were not available.

Facebook’s Marketing API only provides a rounded number of MAUs upon each request. The applied rounding is proportional to the number of MAUs. For example, for values between 1000 and 10,000, the rounding precision is 100; for values between 10,000 and 100,000, the rounding precision is 1000; and so forth. To deal with the rounding mechanism, we estimated the upper and lower bounds of Facebook’s API rounded response. For instance, for a rounded response of 11,000 MAUs, the lower limit is 10,500, and the upper limit is 11,490. We determined these limits by observing the rounded MAU responses of Facebook’s Marketing API and their frequency.

**Fig 3:**
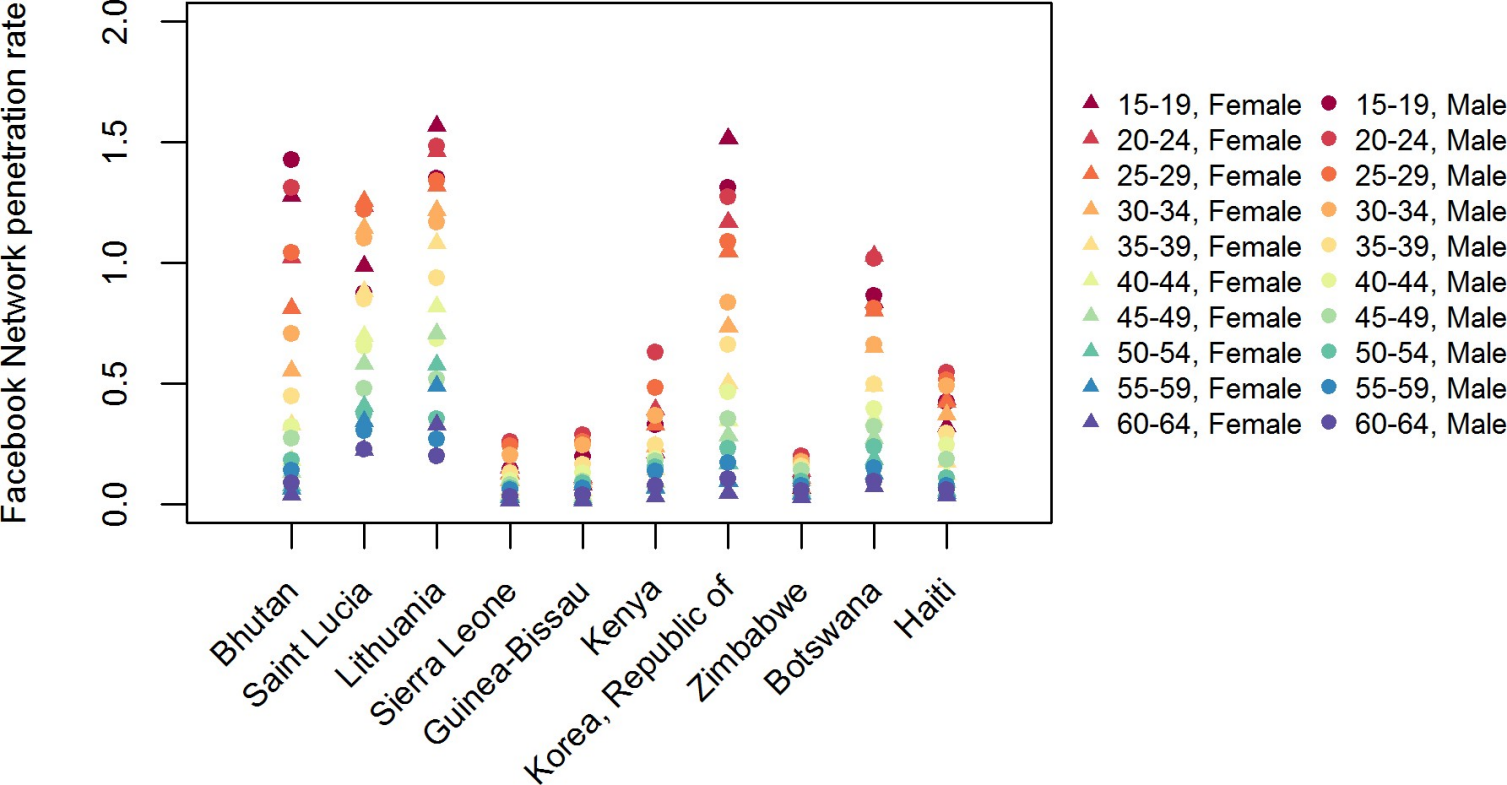
Facebook Network penetration rates *fpr*_*a*,*g*,*c*_ for 10 randomly selected countries by gender (shape) and age group (colour).

Finally, using UNDESA population estimates for 2018 [34], we computed the FN penetration rates. The *fpr*_*a*,*g*,*c*,*p*_ in Eq 6 is the FN penetration rate among individuals in the five-year age group *a*, of gender *g*, in country *c*, and in time *p*. This rate is computed by dividing the *FN_pop_raw*_*a*,*g*,*c*_, which is the number of raw FN users in the five-year age group *a*, of gender *g*, in country *c*, and in time period *p*, by the *UNDESA_pop*_*a*,*s*,*c*_, which is the population estimate provided by UNDESA, and includes both the foreign-born and the native-born population with the same demographic characteristics (same age group a, sex s, country c). UNDESA population statistics are available for 200 countries globally, and are disaggregated by five-year age groups and sex. We used population estimates (medium projection variant) for 2018. As Fig 3 shows, the FN penetration rates vary based on users’ age, gender, and country of residence. Young people are more likely to use the FN than older people.

$$fpr_{a}{}_{,g,c,p}=FN\_pop\_raw_{a,g,c,p}/\;UNDESA\_pop_{a,s,c}$$ (6)

*where a = age*

*s/g = sex/gender* ϵ *[Male*, *Female]*

c = country

*p = data collection period* ϵ [*1*:*(30/1-26/2/2018)*, *2*:*(*13/08-19/9/2018)]

### Model

The main aim of our model was to correct for the potential bias in FN usage by estimating the number of “FN migrants” in the real population. As Fig 3 indicates, the over- or the under-representativeness of FN users varies based on their age and their gender, and on the country under consideration. According to a survey conducted by Smith and Anderson [23], the level of Facebook usage varies based on the users’ origin, income, educational attainment, and area of residence (urban or rural). To correct for the over- or under-representativeness of FN users with respect to the real population, we used a model originally proposed by Spyratos et al. [8]. This approach takes into account the differences in penetration rates by age and gender, both in the country of residence and in the country of previous residence. For instance, it may be assumed that the probability of a male individual aged 20 to 24 who had lived in Kenya and now lives in the US using the FN is affected by the FN penetration of males in the same age group living in Kenya, as well as by the FN penetration rate of males in the same age group residing in the US.

The *FN_mig_cor*^*i*^_*a*,*g*,*o*,*d*,*p*_, in Eq 7 is the corrected number of FN “migrants”, which corresponds to the estimated population in age group *a*, of gender *g*, in time period *p*, who have lived in country o and now live in country *d*. This number is estimated by dividing the raw number of FN migrant users *FN_mig_raw*^*i*^_*a*,*g*,*o*,*d*,*p*_ by the weighted sum of the FN penetration rates in the country of previous residence and the country of current residence of that migrant group. The index *i* in *FN_mig_cor*^*i*^_*a*,*g*,*o*,*d*,*p*_ and *FN_mig_raw*^*i*^_*a*,*g*,*o*,*d*,*p*_ indicates whether the number of the corrected or raw FN migrant users refers to the rounded numbers, to the lower bound, or to the upper bound, as estimated in the FN data preparation section. The weight applied to the FN penetration rate of the country of current residence of a migrant is the *WD*_*a*,*g*,*o*,*d*_, estimated using Eq 8; and the weight applied to the country of previous residence is the *WO*_*a*,*g*,*o*,*d*_, estimated using Eq 9. *WD*_*a*,*g*,*o*,*d*_ reflects the extent to which the FN penetration rate of migrants with a specific age-gender-previous residence assimilate to that of the same age-gender group in the country of current residence. The value that the *WD*_*a*,*g*,*o*,*d*_ may take ranges from zero to one. The value of the *WD*_*a*,*g*,*o*,*d*_ depends on two factors. The first factor is the gender and the age group of the FN users, described by the parameter *z*_*a*,*g*_ ϵ [0,0.1, …,1] in Eq 8. The reason we have chosen to estimate different *z*_*a*,*g*_ for each age group and gender is that–as demonstrated in the next section–people of different genders and ages assimilate to the digital habits of the population in the country of current residence to varying degrees. The second factor–described in Eq 8 –is the difference between the FN penetration rates in the country of current residence and those in the country of previous residence, multiplied by parameter *t* ϵ [0,0.1, …,0.9,1]. We consider the difference between the FN penetration rates in the countries of current and previous residence, since we assume that FN users moving to a country with lower FN use will continue using the FN to stay in contact with their network in their country of previous residence, and therefore adapt to the FN usage patterns in the country of current residence to a lesser degree. By contrast, we assume that migrants moving to a country with higher FN use are more likely to assimilate to the digital habits of the current country of residence in order to stay in contact with their new social and professional network. The estimation of the *z*_*a*,*g*_ and *t* parameters is described in the next section.

$$FN\_mig\_cor^{i}{}_{a,g,o,d,p}=\;FN\_mig\_raw^{i}{}_{a,g,o,d,p}/\left(fpr_{a,g,d,p}*WD_{a,g,o,d}+\;fpr_{a,g,o,p}*\;WO_{a,g,o,d}\right)$$ (7)

*where a* ϵ *[15–19, 20–24, 25–29, 30–34, 35–39, 40–44, 45–49, 50–54, 55–59, 60–64]*

*g* ϵ *[Male*, *Female]*

*o* ⊆ *see S1 Table*

*d* ⊆ *see S2 Table*

*i* ϵ *[r*:*(rounded)*,*l*: *(lower bound)*,*u*:*(upper bound)]*

*p* ϵ *[1*:*(30/1/2018 to 26/2/2018)*, *2*:*(*13/08/2018 to 19/09/2018)]

$$WD_{a,g,o,d}=z_{a,g}+\;t((fpr_{a,g,d}-fpr_{a,g,o})/\;fpr_{a,g,d})$$ (8)

*where z*_*a*,*g*_ ϵ [0,0.1, … 0.9,1]

*t* ϵ [0,0.1, … 0.9,1]

*If WD*_*a*,*g*,*o*,*d*_ *>1 then WD*_*a*,*g*,*o*,*d*_ *= 1*

*If WD_a,g,o,d_* <*0 then WD_a,g,o,d_ = 0*

$$WO_{a,g,o,d}=\;1{-}_{}WD_{a,g,o,d}$$ (9)

### Selection of the optimal weights

For the selection of the optimal values of the *z*_*a*,*g*_ and *t* parameters, which are required for estimating the weight of the current country of residence *WD*_*a*,*g*,*o*,*d*_ in Eq 8, we assessed the correspondence between the number of migrants in the ACS dataset and the corrected rounded number of FN migrants. We used ACS migration statistics for the US instead of UNDESA/OECD statistics, because the UNDESA/OECD statistics are not disaggregated by five-year age groups. Moreover, the use of ACS statistics as a reference will allow us to assess whether the method developed by Spyratos et al. [8] using Eurostat statistics is also valid in a non-EU context. Since the corrected number of FN migrants and the reference migration statistics do not refer to same quantities of interest, we decided against trying to determine whether the two datasets match in absolute numbers. Instead, we look at how closely the datasets are correlated. We therefore used the coefficient of determination *R*^*2*^ to assess how well the corrected number of FN migrants explains the age-gender-previous residence distribution of migrants, as recorded by the ACS.

**Fig 4:**
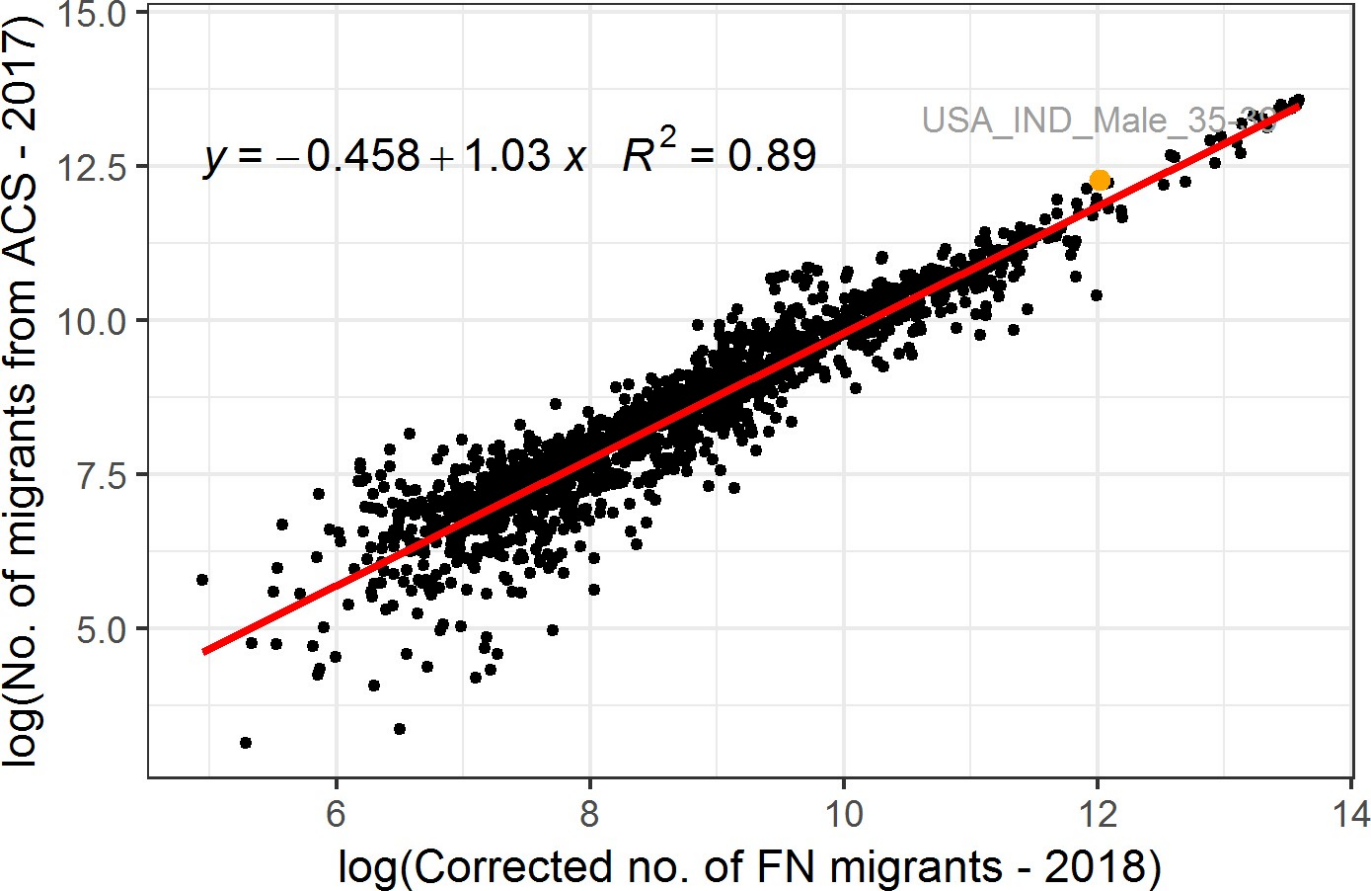
Log-log linear regression between the corrected number of FN migrants and the number of migrants according to the ACS in the US, for each age-gender-country of birth combination. For example, the orange dot represents the number of Indian migrants in the US who are male and belong to the age group 35–39.

We iteratively performed log-log linear regressions–like the one presented in Fig 4 –between the ACS dataset *acs*_*a*,*s*,*o*,*d*_ and different corrected FN datasets *FN_mig_cor*^*i = r*^_*a*,*g*,*o*,*d*,*p = 1*,_ estimated using all possible combinations of *z*_*a*,*g*_ and *t* parameter values. We then selected the log-log linear regression for which we obtained the highest coefficient of determination, the “max *R*^*2*^” column in Table 1. The corrected FN datasets used in the best fitting log-log linear regression represent the most reliable estimates, and the *z*_*a*,*g*_ and *t* parameter values used for their estimation are the optimal values of parameters *z*_*a*,*g*_ and *t* (Table 1). We excluded age-gender-previous residence combinations with less than 1000 migrants in order to reduce the impact of combinations with low numbers of migrants in the model, given that the ACS estimates are based on a sample survey. We also used natural logarithms of both the reference and the FN corrected figures to reduce the influence of age-gender-previous residence combinations with high numbers of migrants in the model. Due to computational limitations, we combined the 10 age groups in the *z*_*a*,*g*_ parameter into four age hyper-groups, as shown in the *a* column of Table 1. We performed four rounds of cross-validation by randomly selecting 51 countries of birth as the training data subset used for estimating the optimal *z*_*a*,*g*_ and *t* parameter values, and the remaining 25 countries of birth as the testing data subset. For the calculation of the testing *FN_mig_cor*^*i = r*^_*g*,*o*,*d*,*p = 1*_ subset, we used the *z*_*a*,*g*_ and *t* parameter values found using the training subset of the same cross-validation round. As Table 1 shows, the *R*^*2*^ values of the correlation between the training subsets, the testing subsets, and the reference statistics are both high and similar; thus, we assume that the proposed model can be generalised to the US.

**Table 1:** *z*_*a*,*g*_ and *t* parameter values for which the highest *R*^*2*^ values were obtained when correlating the training subsets of the *log(FN_mig_cor^i = r^_a_*_,*g*,*o*,*d*,*p = 1*_*)* and the *log(acs*_*a*,*s*,*o*,*d*_*)*. We performed four rounds of cross-validation by randomly selecting in each round 51 countries of birth as the training subset and the remaining 25 countries of birth as the testing subset.

	*g*	*a*	Training subsets, *51 countries of birth in the US*				Median	Training with Eurostat [8]
			*1a*	*2a*	*3a*	*4a*		
*z*_*a*,*g*_	female	15–24	0.8	1	0.9	0.8	**0.85**	**0.85**
		25–39	1	0.8	0.7	0.7	**0.75**	**0.5**
		40–54	0.1	0.3	0.4	0.3	**0.3**	**0.3**
		55–64	0	0.3	0.3	0.2	**0.25**	**0.3**
	male	15–24	0.7	1	0.9	0.9	**0.9**	**0.95**
		25–39	1	1	0.8	1	**1**	**0.8**
		40–54	0.4	0.6	0.6	0.6	**0.6**	**0.8**
		55–64	0.5	0.9	0.6	0.6	**0.6**	**0.85**
*t*			0.6	0.2	0.2	0.3	**0.25**	**0.25**
*Training Max R*^*2*^			0.92	0.90	0.90	0.91	**0.91**	**0.83**
			**Testing subsets**, *25 countries of origin in the US*					
			*1b*	*2b*	*3b*	*4b*		
*Testing R*^*2*^			0.87	0.90	0.90	0.86		

The optimal parameter values found by this study using ACS statistics are the median values of the four training subsets, and are highlighted in the black box in Table 1. As Table 1 indicates, the optimal parameter values found in this study and by Spyratos et al. [8] using Eurostat migration statistics (also in Table 1) are similar, which demonstrates the robustness of the proposed model. Male FN migrants aged 40–64 are shown to assimilate to the FN penetration rate of their current country of residence to a greater extent than female FN migrants in the same age group. Second, FN migrants in the younger age groups appear to assimilate to the FN penetration rate of their current country to a greater extent than than FN migrants aged 40–64. Differences are observed in the *z*_*a*,*g*_ parameter values of females aged 25–39 and males aged 40–64. The values of the coefficient of determination *R*^*2*^ are higher when the FN estimates are compared to the ACS figures than when they are compared to the Eurostat figures. The ACS is a representative survey of 3.5 million US households organised by the U.S. Census Bureau. But since Eurostat migration statistics are collected by the various EU Member States, and the methodologies for collecting these data differ across countries, discrepancies between these datasets are possible.

**Fig 5:**
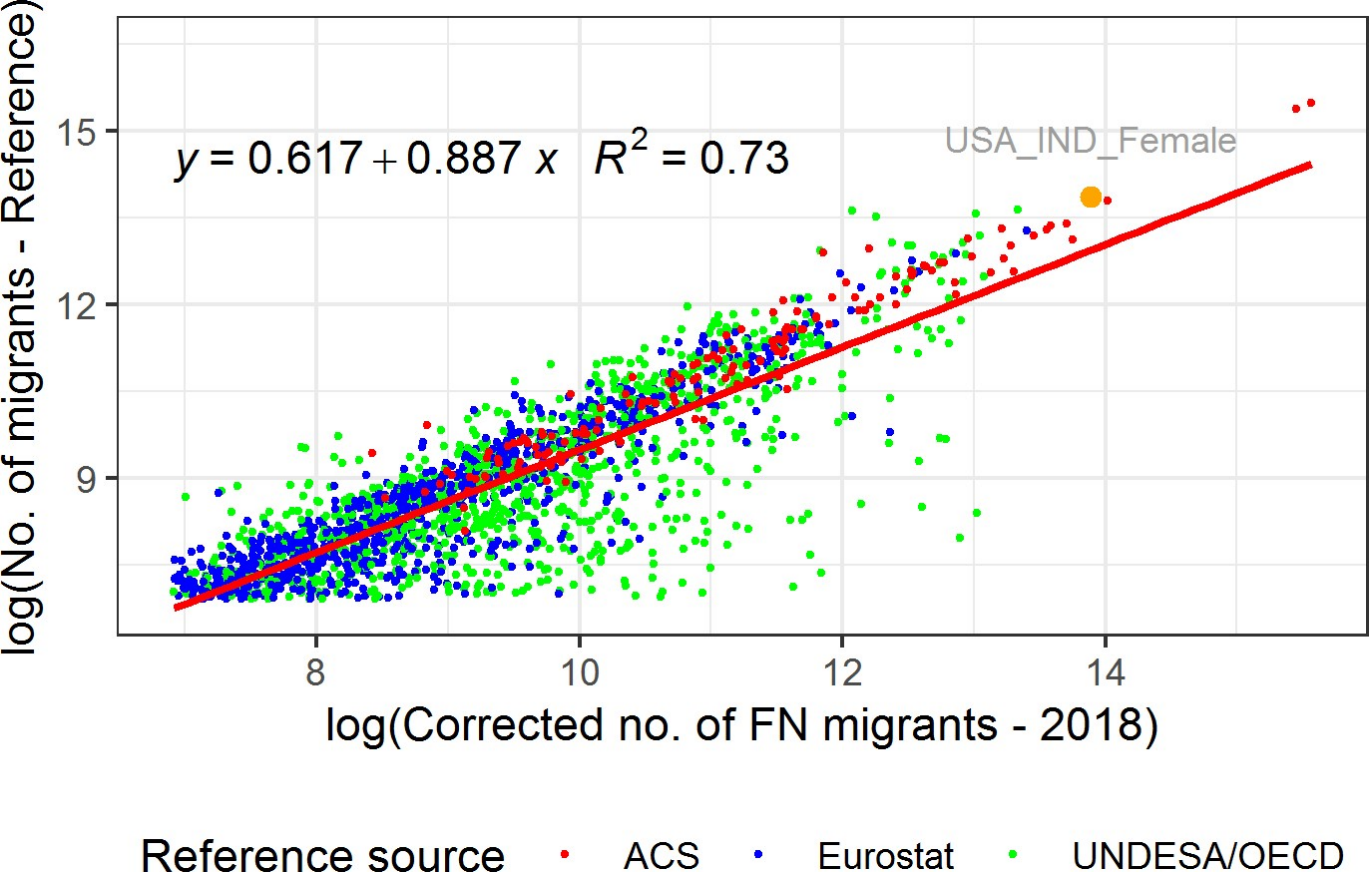
Log-log linear regression between the corrected number of FN migrants and the number of migrants from all the reference sources aged 15–64 for each gender, country of previous residence, and current residence combination with more than 1000 migrants. For example, the orange dot represents the Indian migrants in the US who are female and belong to the age group 15–64.

As Fig 4 shows, the corrected number of FN migrant users estimated using optimal *z*_*a*,*g*_ and *t* parameter values in the US, for each five-year age group, gender, and previous residence combination, are strongly correlated with the ACS reference statistics (*R*^*2*^ = 0.89, *p* <0.001). The estimated number of FN migrant users aged 15–64 for each gender, previous residence, and current residence combination using the optimal *z*_*a*,*g*_ and *t* parameter values found in this study is strongly correlated (*R*^*2*^ *=* 0.734, *p <*0.001) with the number of international migrants provided by official sources (see Fig 5 in next section). The same correlation using the *z*_*a*,*g*_ and *t* parameter values found in Spyratos et al. [8] is similar (*R*^*2*^ *=* 0.733, *p <*0.001).

## Results

### Analysis of January/February 2018 Facebook Network data

The corrected numbers of FN migrants aged 15–64 for each gender, country of previous residence, and country of current residence are strongly correlated with the migrant stock numbers provided by official sources (*R*^*2*^ *=* 0.735, *p <*0.001) (Fig 5). The red dots in Fig 5 represent the corrected number of FN migrants and are compared to the ACS statistics, while the blue dots are compared to the Eurostat statistics, and the green dots are compared to the UNDESA/OECD statistics. There are 2005 combinations of gender, country of previous residence, and current residence when there are more than 1000 migrants in the FN and the reference migration statistics. For 42% of these 2005 combinations, the corrected number of FN migrants deviate from the reference migration statistics within a range of +-30%. When the FN-derived estimates are compared with the ACS statistics, the percentage of observations within a +-30% difference range is 58%. The corresponding figure for the comparison with the Eurostat statistics is 50%, while the corresponding figure for the comparison with the UNDESA/OECD statistics is only 32%.

Table 2 shows the 25 previous and current residence combinations with the highest number of corrected FN migrants, and the equivalent number of migrants according to the reference statistics. As Table 2 indicates, the corrected number of FN migrants from the US in Indonesia is overestimated by 11,520% compared to the UNDESA/OECD figures. This overestimation may be due to fake accounts linked to “click farms” based in Indonesia [35]. The methodology also leads to an overestimation by 2092% of the number of Venezuelan migrants in Colombia. According to Colombian migration authorities, there were 600,000 Venezuelans in Colombia as of February 2018 [36], and this number did not include Venezuelans with dual citizenship or their Venezuelan-born children. In this case, the figures reported in the official statistics may under-represent the actual figures. Thus, our estimate of 699,537 FN Venezuelan migrants aged 15–64 in Colombia seems reasonable. The corrected number of FN Filipino migrants aged 15–64 is overestimated by 273% in Japan and by 90% in Canada, and there are no indications that such an overestimation may be valid. As Table 2 shows, the FN migrants from Guatemala and Honduras in the US are overestimated by 75% and 93%, respectively, compared to the ACS-provided figures. It is worth mentioning that in the second data collection period, which is described later in this section, the numbers of FN migrants from Guatemala and Honduras in the US are downgraded by 11% and 12%, respectively. This overestimation and the significant decrease in the FN-derived raw and corrected numbers within a six-month period raises concerns about whether FN-provided figures can be re-used for migration statistics. However, the figures provided by the FN for migrants from Jamaica, the United Kingdom, Canada, and India are very similar to those provided by the ACS (+-1% difference).

**Table 2:** The 25 previous-current residence combinations with the highest corrected number of migrants from the FN and the corresponding number of migrants from official sources (UNDESA/OECD & Eurostat & ACS). All numbers are in thousands.

Current residence	Previous residence	FN raw	FN corrected			Reference	Difference (FN cor. ro.- ref.)	
			rounded	lower	upper		No	%
**United States**	Mexico	10,660	10,890	10,769	11,011	10,083	808	8%
**United States**	India	2,006	2,196	2,155	2,249	2,196	0	0%
**United States**	Philippines	1,752	2,119	2,076	2,172	1,625	494	30%
**United States**	El Salvador	1,519	1,564	1,532	1,605	1,226	338	28%
**United States**	Guatemala	1,521	1,495	1,463	1,527	853	643	75%
**United States**	Dominican R.	1,173	1,283	1,270	1,295	978	305	31%
**United States**	Honduras	1,073	1,103	1,085	1,121	571	532	93%
**Germany**	Poland	613	1,065	1,044	1,087	1,616	-551	-34%
**Italy**	Romania	871	1,051	1,039	1,064	968	83	9%
**United States**	Vietnam	803	972	959	984	1,107	-135	-12%
**France**	Algeria	699	872	857	887	1,001	-130	-13%
**Indonesia**	United States	1,092	851	826	876	7	844	11520%
**Canada**	Philippines	697	844	831	858	444	401	90%
**Japan**	Philippines	468	769	753	785	206	563	273%
**United States**	Brazil	763	767	756	779	427	341	80%
**United Kingdom**	Poland	760	765	757	774	749	16	2%
**United States**	Colombia	666	759	746	771	665	94	14%
**Canada**	India	644	752	740	764	483	269	56%
**France**	Morocco	593	703	692	716	778	-74	-10%
**Colombia**	Venezuela	709	700	690	709	32	668	2092%
**United States**	Canada	597	646	634	657	648	-2	-0%
**United Kingdom**	India	540	642	631	653	637	5	1%
**Canada**	United States	588	610	598	621	202	408	202%
**United States**	Jamaica	515	597	586	609	601	-3	-1%
**United States**	United Kingdom	562	587	576	598	592	-5	-1%

**Fig 6:**
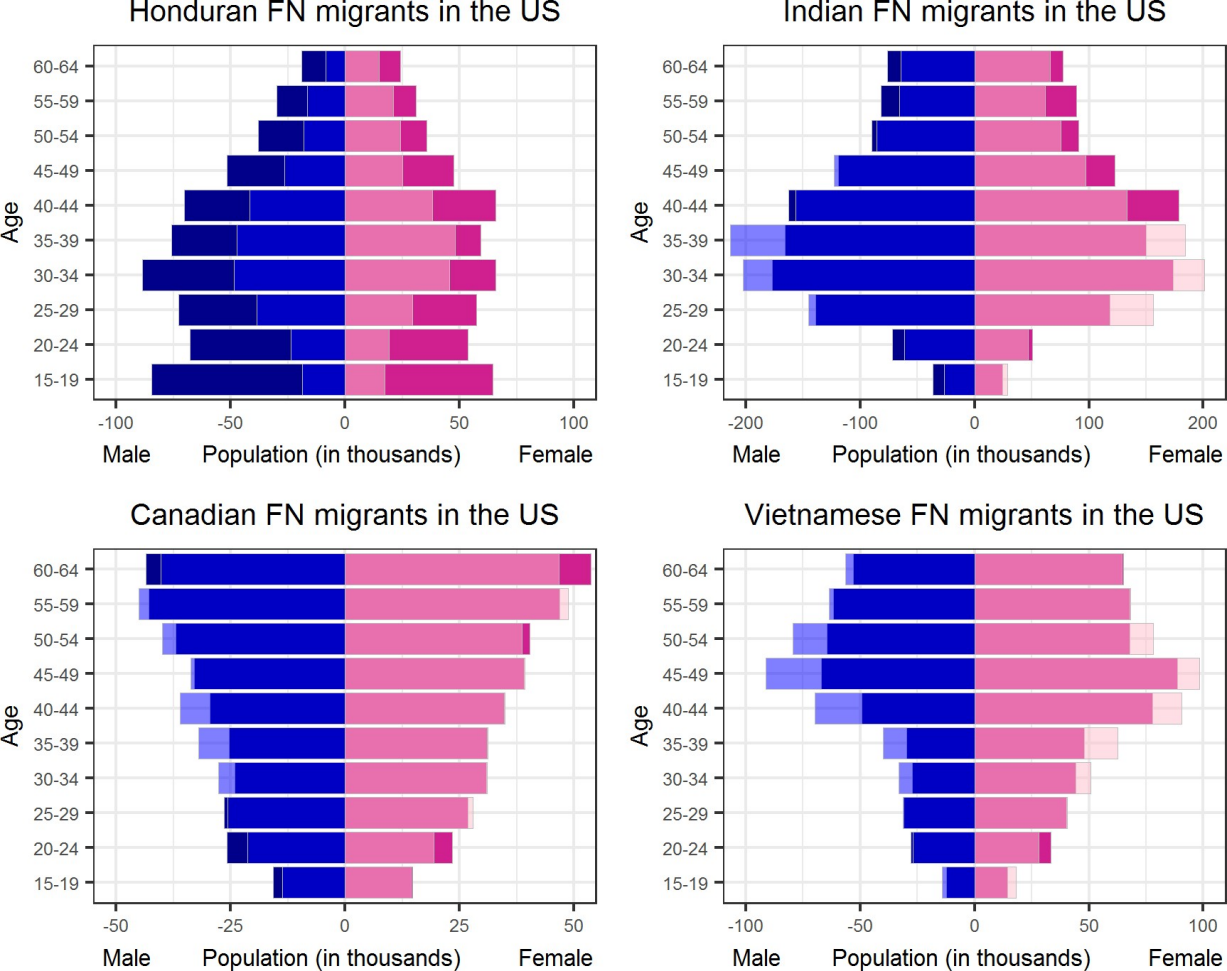
Population pyramids for four countries of previous residence in the US. Light shading = ACS, mid shading = ACS & corrected FN, and dark shading = corrected FN.

Fig 6 displays the population pyramids of the corrected numbers of Honduran, Indian, Canadian, and Vietnamese FN migrants in the US. As Fig 6 shows, using FN migrant statistics allows us to accurately estimate the age-gender distribution of Canadian FN migrants in the US, but leads us to overestimate the number of Honduran FN migrants, and particularly those in the age group 15–19. The analysis of the differences between the corrected numbers of FN migrants and the numbers of international migrants from official sources can reveal trends that do not yet appear in the official statistics. However, additional knowledge is needed to verify whether an increasing trend is due to a real migration flow, or to FN-related factors, like the existence of fake accounts or changes in the classification algorithms of Facebook’s advertising platform.

### Comparative analysis between January-February 2018 & August-September 2018

This section provides a comparative analysis of the corrected number of FN migrants between two time periods. The first data collection period lasted from 30/1/2018 to 26/2/2018 and the second lasted from 13/08/2018 to 19/09/2018. Using Eq (7), we estimated the corrected number of FN migrants for the two time periods by using the same *z*_*a*,*g*_ and *t* parameter values and the FN raw number of migrant users (*FN_mig_raw*^*i*^_*a*,*g*,*o*,*d*,*p*_) and total users (*FN_pop_raw*_*a*,*g*,*c*,*p*_), which correspond to each time period *p = 1* and *p =* 2. The updated numbers of total FN users of age *a*, gender *g*, in country *c* (*FN_pop_raw*_*a*,*g*,*c*,*p = 2*_) were used for the estimation of the updated FN penetration rates (*fpr*_*a*,*g*,*c*,*p* = 2_). Because the minimum MAU response of Facebook’s Marketing API to queries was increased on 26/2/2018 from 20 to 1000 users, only gender-5-year age groups with more than 1000 migrants were taken into account for this comparison.

**Fig 7:**
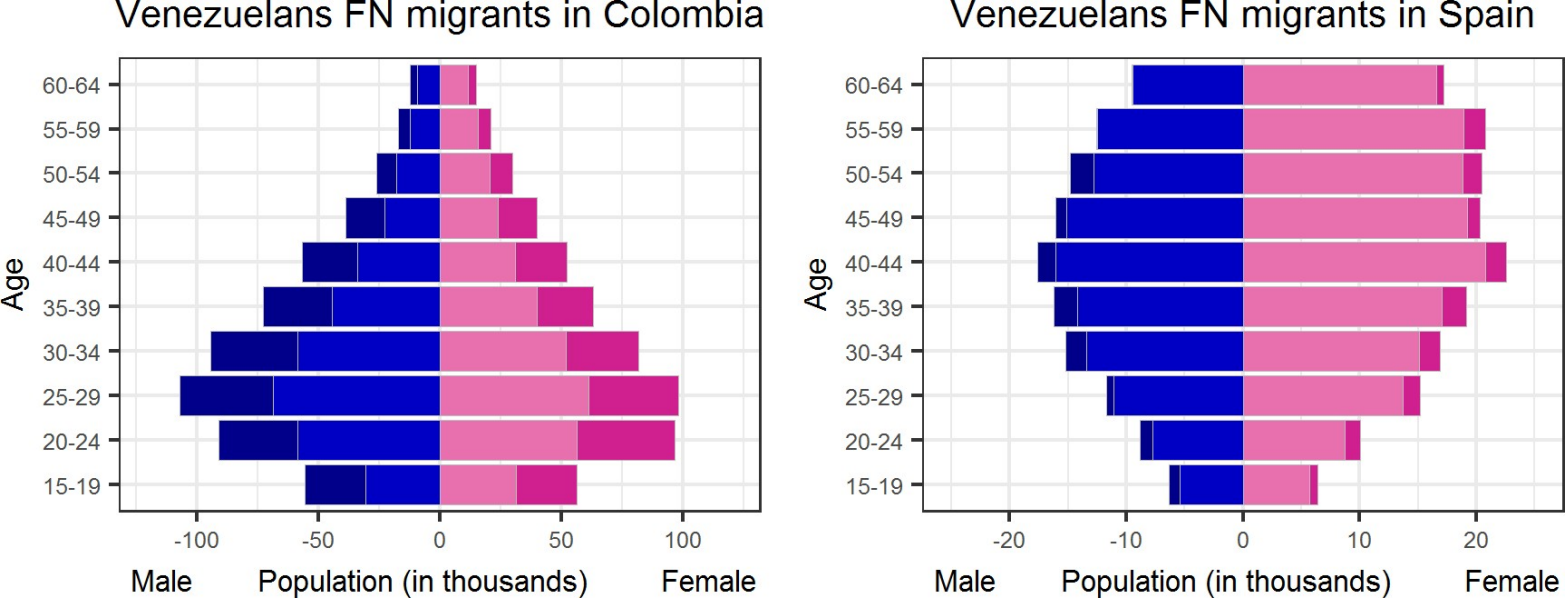
Population pyramids of the corrected FN migrants from Venezuela in Colombia (left side) and in Spain (right). Light shading = January-February 2018 population, mid shading = January-February 2018 & August-September 2018, and dark shading = August-September 2018.

Table 3 presents the results of this comparison. A significant increase (+62%) is observed in the corrected number of Venezuelan FN migrants aged 15–64 in Colombia: i.e., from 699,548 in February 2018 to 1,156,300 in August 2018. According to Colombian migration authorities, the number of Venezuelan migrants of all ages in Colombia was 600,000 (not including Venezuelans with dual citizenship or their Venezuela-born children) in February 2018 [36], and was close to 1,000,000 in August 2018 [37]. This means that the FN data would have enabled us to anticipate the increase in the number of Venezuelans in Colombia–and, importantly, as the left side of Fig 7 shows, to have identified their age-gender distribution. As Fig 7 reveals, the “recently” arrived Venezuelan FN migrants in Colombia are young, while the “recently” arrived Venezuelan FN migrants in Spain are middle-aged. Furthermore, as Table 3 indicates, both the corrected and the raw number of FN migrants from Latin America countries in the US decreased. As we mentioned in the previous section, the FN migrants from Latin American countries in the US are overestimated; thus, this decrease may represent a partial correction of the FN’s overestimated figures.

**Table 3:** Difference between the corrected number of FN migrants in January-February 2018 and in August-September 2018, for the 15 previous-current residence combinations with higher corrected numbers of FN migrants. All numbers are in thousands.

Current residence	Previous residence	August-September		January—February		Difference Cor.	
		Raw	Corrected	Raw	Corrected	No	%
United States	Mexico	9,970	10,330	10,660	10,890	-560	-5%
United Arab Emirates	India	2,724	2,608	2,886	2,606	2	0%
United States	India	2,013	2,274	2,006	2,196	77	4%
United States	Philippines	1,676	2,031	1,752	2,119	-88	-4%
United States	Puerto Rico	1,481	1,533	1,595	1,653	-120	-7%
United States	El Salvador	1,374	1,463	1,519	1,564	-101	-6%
United States	Guatemala	1,300	1,334	1,521	1,495	-161	-11%
United States	Dominican Rep.	1,150	1,262	1,173	1,283	-20	-2%
Colombia	Venezuela	1,156	1,126	709	700	426	**61%**
Germany	Poland	660	1,110	613	1,065	44	4%
Italy	Romania	845	1,035	871	1,051	-16	-2%
United States	Viet Nam	790	975	803	972	3	0%
United States	Honduras	919	971	1,073	1,103	-132	-12%
United Arab Emirates	Philippines	1,065	945	1,149	988	-43	-4%
Canada	Philippines	698	834	697	844	-10	-1%

## Discussion and conclusions

In this article, we estimated the number of FN migrants in 55 countries after correcting for sample bias; we compared these estimates with official migration statistics; and we performed a comparative analysis of the number of estimated FN migrants for two separate time periods. The FN’s criteria for classifying a user as having “lived in country X” are not very detailed, and they might change over time. Thus, the main limitation of this study is the lack of information on how FN classifies its users as “having lived in country X”, and on how accurately it does so. The existence of a large number of fake accounts might also distort the FN-provided figures. Moreover, the main limitation of studying the proportional increase or decrease of FN migrants over time is that the FN can change its classification algorithms at any time, without prior notice. For example, in mid-March 2019, the FN changed the way that it was classifying users as having “lived in country X”; and this change resulted in significantly lower FN-provided figures.

Although the the FN’s definition of users who “lived in country X” is similar to the criterion of previous residence, because of the lack of availability of international migrant stock statistics by previous residence, we decided to compare FN-derived estimates with international migrant stock statistics by country of birth. This comparison of traditional and non-traditional statistics resulted in the following discrepancies. First, as we noted above, the definition of FN migrants is not precisely known, and it clearly differs from the standardised definitions of migrants used in international statistics. For example, the minimum amount of time a person has to spend abroad to be classified as a FN user who had lived abroad (FN migrant) is unknown, whereas the UN-recommended definition of an international migrant, which is used in Eurostat and UNDESA statistics, requires an individual to have stayed in the country of current residence for at least 12 months to be classified as migrant [29]. Second, FN users can be classified as having lived in more than one country as long as the algorithms of Facebook’s advertising platform detect that they lived in those countries in the past, while a migrant can be recorded only once in the international migrant stock statistics by country of birth, which are used as a reference in this study. Finally, official statistics are normally disaggregated by sex based on individuals’ biological characteristics, while FN data are disaggregated by gender based on how users identify themselves.

To correct for the bias in the FN sample, we used an extension of the method originally proposed by Spyratos et al. [8]. The aim of this method was to generate independent estimates of FN migrants by calibrating the raw number of FN migrants based on the penetration rate of the FN in the country of current residence, and in the country of previous residence of a given FN user. The calculation of the FN penetration rate in each country was based on figures provided by the FN itself and on population data. The proposed method used migration statistics only in order to identify the degree to which a migrant assimilates to the FN usage patterns of the destination country. As Table 1 shows, the methodology proved to be robust, as it resulted parameter values similar to those found when using both the ACS and the Eurostat migration reference statistics.

The corrected number of FN migrants was strongly correlated (*R*^*2*^ *=* 0.735, *p <*0.001) with migrant stock figures from official statistics. The correlation was even higher when the FN-derived estimates of migrants were compared with the ACS-derived estimates for the US (*R*^*2*^ *=* 0.887, *p <*0.001). However, as we described in the results section, although the numbers of FN migrants from some countries of previous residence in the US were very close to the ACS estimates, for other countries of previous residence–and especially for Latin American countries–the numbers of FN migrants were significantly higher than the numbers of migrants from these countries in the US. Other inconsistencies of the FN-provided figures include the significant undercounting of Greek migrants worldwide and the over-counting of US migrants in Indonesia.

**Fig 8:**
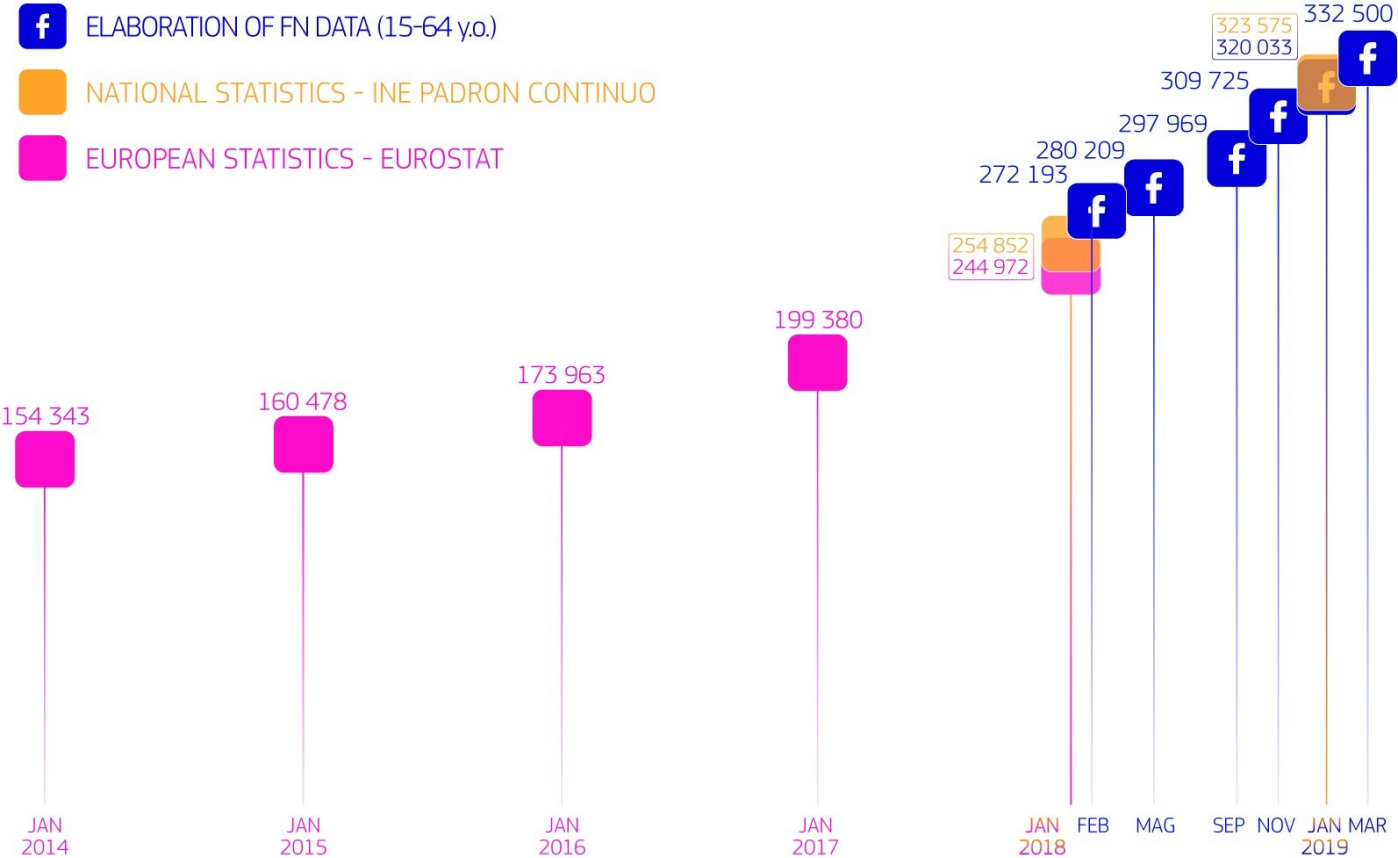
Stocks of international migrants and FN migrants from Venezuela in Spain. The national and Eurostat statistics refer to all ages, while the FN-derived estimates refer to ages 15–64.

The discrepancies that could be observed in the comparison of the FN estimates and the official statistics may represent, apart from non-addressed problems related to the correction of sample bias or to the FN-provided raw figures, useful indications of emerging trends that have not yet been captured in the official statistics. Such trends were, for example, detected for Venezuelan migrants in Colombia and Spain. In the case of Colombia, we used FN data to successfully identify and quantify an increase in the stock of migrants, which had been anecdotally reported in several other sources of information, but which had not yet appeared in the official statistics. As Fig 8 shows, we used FN data to anticipate the increase in Venezuelans in Spain, and this trend had since been verified by the 2019 statistics from the National Statistical Office of Spain [38]. Using FN-derived statistics, we correctly captured the increase in Venezuelans in Spain in 2019, but we overestimated their number, since our estimations refer to the population aged 15–64, while the INE statistics in Fig 8 refer to migrants of all ages.

Due to the uncertainties associated with using FN-provided figures, FN-derived migrant estimates should not be seen as replacements for official migration statistics. Nevertheless, as long as they can be validated using reliable third-party data, these additional estimates have great value for at least three main reasons. First, these estimates can anticipate emerging trends and support early-warning mechanisms that facilitate the monitoring of migration at the national, sub-national, and local levels. Second, these estimates can provide a finer description of the characteristics of the migrant population. Third, these estimates can offer a wider definition of migrants that captures forms of migration that do not appear in the official statistics. The validation of emerging migration trends that are estimated using third-party migration data from non-traditional sources is essential. Even after correcting for a sample bias or eliminating any uncertainties associated with classifying the users as “migrants”, analysing social media data to inform policy decisions has several limitations. As the social media companies that provide the data are commercial enterprises, questions regarding the strategic autonomy and independence of such approaches arise when the data are being re-used, not for advertising purposes, but to provide input for policy-making. At any time, social media companies may change the conditions for accessing the data or decide to change the types of available variables, and their definitions. Moreover, third parties might try to manipulate FN content to promote their political agenda. For example, anti-immigrant interest groups may be able to influence the public debate on migration by increasing the number of FN “migrants” from specific backgrounds through the creation of fake FN accounts.

With respect to big data, the re-use of pre-aggregated figures by Facebook’s advertising platform, comes at the price of having limited control over how the figures are processed and aggregated. However, an advantage of using these data for researchers is that they do not have to deal with the legal and technical issues they normally face when analysing large volumes of individual records. We can therefore conclude that the systematic use of non-traditional data for policy support and migration governance will require researchers to negotiate agreements with social media companies that provide them with more control over how the data is being produced and pre-processed.

## Supporting information

S1 Table List of countries of previous residence for which FN provides user statistics. (DOCX)

S2 Table List of countries of current residence for which we collected FN user statistics. (DOCX)
