# Supplementary material for: Quantifying international human mobility patterns using Facebook Network data
Source: PLoS One. 2019 Oct 24;14(10):e0224134. doi: 10.1371/journal.pone.0224134 (PMC6812739; doi:10.1371/journal.pone.0224134)
Supplement: S2 Table — (DOCX) [file pone.0224134.s002.docx]

**S2 Table. List of countries of current residence for which we harvested FN user statistics.** We used reference migration data for 55 countries of current residence, which are highlighted in red (ACS), green (UNDESA/OECD), and blue (Eurostat).

| 1 | Afghanistan | 41 | Cote d'Ivoire | 81 | Jordan |
| --- | --- | --- | --- | --- | --- |
| 2 | Albania | 42 | Croatia | 82 | Kazakhstan |
| 3 | Algeria | 43 | Curacao | 83 | Kenya |
| 4 | Angola | 44 | Cyprus | 84 | Kiribati |
| 5 | Antigua and Barbuda | 45 | Czech Republic | 85 | Korea, Republic of |
| 6 | Argentina | 46 | Denmark | 86 | Kuwait |
| 7 | Armenia | 47 | Djibouti | 87 | Kyrgyzstan |
| 8 | Aruba | 48 | Dominican Republic | 88 | Lao People's Democratic Republic |
| 9 | Australia | 49 | Ecuador | 89 | Latvia |
| 10 | Austria | 50 | Egypt | 90 | Lebanon |
| 11 | Azerbaijan | 51 | Equatorial Guinea | 91 | Lesotho |
| 12 | Bahamas | 52 | Eritrea | 92 | Liberia |
| 13 | Bahrain | 53 | Estonia | 93 | Libya |
| 14 | Bangladesh | 54 | Ethiopia | 94 | Lithuania |
| 15 | Barbados | 55 | Fiji | 95 | Luxembourg |
| 16 | Belarus | 56 | Finland | 96 | Macao |
| 17 | Belgium | 57 | France | 97 | FYROM |
| 18 | Belize | 58 | Gambia | 98 | Madagascar |
| 19 | Benin | 59 | Germany | 99 | Mali |
| 20 | Bhutan | 60 | Ghana | 100 | Malta |
| 21 | Bolivia | 61 | Greece | 101 | Moldova, Republic of |
| 22 | Bosnia and Herzegovina | 62 | Guadeloupe | 102 | Mongolia |
| 23 | Botswana | 63 | Guam | 103 | Montenegro |
| 24 | Brazil | 64 | Guatemala | 104 | Morocco |
| 25 | Brunei Darussalam | 65 | Guinea | 105 | Myanmar |
| 26 | Bulgaria | 66 | Guinea-Bissau | 106 | Netherlands |
| 27 | Burkina Faso | 67 | Guyana | 107 | Poland |
| 28 | Burundi | 68 | Haiti | 108 | Portugal |
| 29 | Cambodia | 69 | Honduras | 109 | Romania |
| 30 | Cameroon | 70 | Hong Kong | 110 | Saint Lucia |
| 31 | Canada | 71 | Hungary | 111 | Slovakia |
| 32 | Cape Verde | 72 | Iceland | 112 | Slovenia |
| 33 | Central African Republic | 73 | India | 113 | Spain |
| 34 | Chile | 74 | Indonesia | 114 | Sri Lanka |
| 35 | China | 75 | Iraq | 115 | Sweden |
| 36 | Colombia | 76 | Ireland | 116 | Switzerland |
| 37 | Comoros | 77 | Israel | 117 | United Arab Emirates |
| 38 | Congo | 78 | Italy | 118 | United Kingdom |
| 39 | Congo DCR | 79 | Jamaica | 119 | United States |
| 40 | Costa Rica | 80 | Japan | 120 | Western Sahara |
|  |  |  |  |  |  |
|  | Removed in the data preparation stage |  | Eurostat |  | UNDESA/OECD |
|  |  |  |  |  |  |
|  | ACS |  |  |  |  |
